# Reductive Sn^2+^ Compensator for Efficient and Stable Sn‐Pb Mixed Perovskite Solar Cells

**DOI:** 10.1002/advs.202400962

**Published:** 2024-04-18

**Authors:** Qiuxiang Wang, Jiaxing Xiong, Yanjun Xing, Xinlei Gan, Wendong Zhu, Rong Xuan, Xiaohui Liu, Like Huang, Yuejin Zhu, Jing Zhang

**Affiliations:** ^1^ School of Physical Sciences and Technology Ningbo University Ningbo 315211 China; ^2^ College of Science and Technology Ningbo University Ningbo 315300 People's Republic of China

**Keywords:** defect passivation, energy level alignment, perovskite solar cells, reductive Sn^2+^ compensator, tin‐lead

## Abstract

Tin‐lead (Sn‐Pb) mixed perovskite with a narrow bandgap is an ideal candidate for single‐junction solar cells approaching the Shockley‐Queisser limit. However, due to the easy oxidation of Sn^2+^, the efficiency and stability of Sn‐Pb mixed perovskite solar cells (PSCs) still lag far behind that of Pb‐based solar cells. Herein, highly efficient and stable FA_0.5_MA_0.5_Pb_0.5_Sn_0.5_I_0.47_Br_0.03_ compositional PSCs are achieved by introducing an appropriate amount of multifunctional Tin (II) oxalate (SnC_2_O_4_). SnC_2_O_4_ with compensative Sn^2+^ and reductive oxalate group C_2_O_4_^2−^ effectively passivates the cation and anion defects simultaneously, thereby leading to more n‐type perovskite films. Benefitting from the energy level alignment and the suppression of bulk nonradiative recombination, the Sn‐Pb mixed perovskite solar cell treated with SnC_2_O_4_ achieves a power conversion efficiency of 21.43%. More importantly, chemically reductive C_2_O_4_^2−^ effectively suppresses the notorious oxidation of Sn^2+^, leading to significant enhancement in stability. Particularly, it dramatically improves light stability.

## Graphical abstract

Herein, highly efficient mixed Sn‐Pb PSCs are achieved by introducing multifunctional Tin (II) oxalate (SnC_2_O_4_). SnC_2_O_4_ with compensative Sn^2+^ and passivate defect, reduce Sn^4+^ and eliminate SnF_2_ and PbI_2_ residues. The device treats with SnC_2_O_4_ achieve a PCE of 21.43%. More importantly, chemically reductive C_2_O_4_^2‐^ effectively suppresses the oxidation of Sn^2+^, leading to significant enhancement on stability.

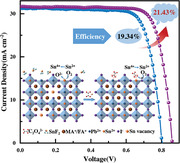

## 1 Introduction

Organic‐inorganic hybrid perovskite solar cells (OIH‐PSCs) have attracted widespread attention due to their excellent photoelectric performance, simple solution processing technology, and wide applications.^[^ ^1^ ^]^ In the past decades, tremendous research efforts have rapidly propelled the power conversion efficiency (PCE) from 3.8% to 26.1%.^[^ ^2^ ^]^ Introducing Sn to partially replace the similar ion radius Pb results in relatively high carrier mobility and a nearly ideal bandgap, which is expected to reach the maximum PCE of 33% based on the Shockley‐Queisser (S‐Q) limit theory.^[^ ^3^ ^]^ In addition, tin‐lead (Sn‐Pb) mixed perovskite with tunable bandgap can be used for all perovskite tandem devices, which is expected to achieve PCE exceeding the S‐Q limit.^[^ ^4^ ^]^ However, the easy oxidation of Sn^2+^ to Sn^4+^ leads to p‐type self‐doping and short carrier lifetimes, resulting in a decrease in device performance.^[^ ^5^ ^]^ Therefore, the preparation of efficient and stable Sn‐Pb mixed PSCs remains challenging.^[^ ^6^ ^]^

To restrain Sn^2+^ oxidation, and thus improve the performance and stability of Sn‐Pb mixed PSCs, extensive research has been conducted. It was first reported in 2016 that a Sn compensation agent was added to the Sn‐Pb precursor solution.^[^ ^7^ ^]^ Since then, SnF_2_ has been widely used as a basic Sn compensation agent in research on Sn‐Pb mixed PSCs.^[^ ^4^, ^8^ ^]^ Density functional theory (DFT) calculations indicated that Sn‐rich environments can effectively increase the formation energy of Sn vacancies (V_Sn_), thereby reducing the defect density of thin films.^[^ ^9^ ^]^ The loss of Sn^2+^ originating from oxidation or intrinsic V_Sn_ formation can be compensated for by SnF_2_.^[^ ^10^ ^]^ Other Sn halides, namely SnCl_2_, SnBr_2_, and SnI_2_, also have similar effects.^[^ ^11^ ^]^ However, excess SnF_2_ presents as a second phase that is prone to aggregate at the grain boundaries or surfaces of the perovskite films, therefore deteriorating the film morphology.^[^ ^12^ ^]^ F^−^ ions preferentially accumulate at the hole transport layer/perovskite interface with high SnF_2_ content, leading to more defects that hinder hole transport.^[^ ^10^ ^]^ Besides the Sn^2+^ compensation methodology, reductive agents have been added to the Sn‐Pb mixed perovskite precursor solution to realize Sn^2+^ stabilization.^[^ ^13^ ^]^ Tin powder precursor additive,^[8a]^ a zwitterionic antioxidant Formamidine sulfinic acid (FSA)^.[^ ^1a^ ^]^ and phenylhydrazinium cation^[^ ^14^ ^],^ etc. are reported to effectively reduce the Sn^4+^ to Sn^2+^, restricting V_Sn_ in perovskite to achieve enhanced performance and stability. Moreover, the Lewis base coordination strategies are also applied successfully to passivate the undercoordinate Sn/Pb to stabilize the Sn^2+^/Pb^2+^.^[^ ^15^ ^]^ Though many materials have been discovered and used by researchers to inhibit Sn^2+^ oxidation, multifunctional additives to simultaneously achieve Sn^2+^ compensation, reduction of Sn^4+^, and passivation of undercoordinate Sn/Pb defects should be explored to realize perfect device performance.

In this work, we report a multifunctional additive Tin (II) oxalate (SnC_2_O_4_), as an additive in the precursor solution. Bivalent Sn^2+^ ions offset part of V_Sn_ and C_2_O_4_^2−^ form coordination bonds with Pb^2+^/Sn^2+^ (C═O─Pb/ C═O─Sn). Therefore, the cation and anion defects are simultaneously passivated, which effectively suppresses the bulk non‐radiative recombination. Furthermore, Sn^4+^ is reduced to Sn^2+^ by reductive oxalic acid groups, which inhibits the generation of V_Sn_ and improves the device's performance. With this multifunctional additive, the resulting perovskite films show excellent surface morphology, low defect density, and outstanding electrical properties. Consequently, with 4% optimal SnC_2_O_4_ addition, the PCE of FA_0.5_MA_0.5_Pb_0.5_Sn_0.5_I_0.47_Br_0.03_ perovskite device is enhanced to 21.43%. More importantly, the long‐term stability and light stability of the device is greatly improved because the chemically reduced oxalic acid groups can effectively inhibit the oxidation of Sn^2+^ ions.

## 2 Results and Discussion

**Figure 1:**
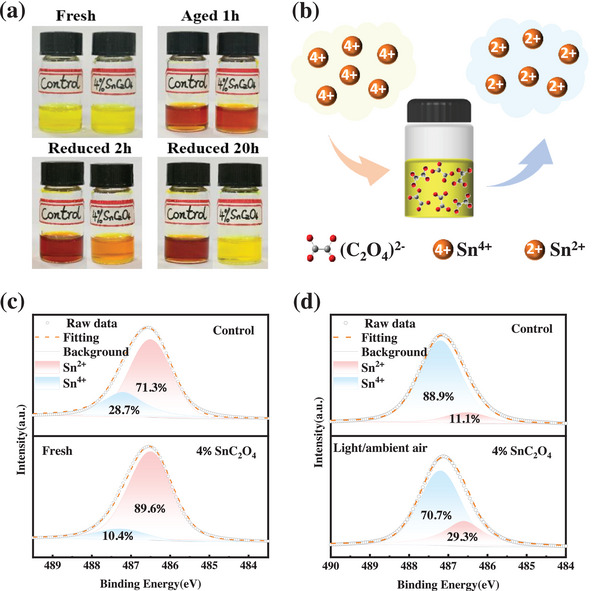
a) Photographs illustrating the facile oxidation of Sn^2+^ to Sn^4+^ in air and the reduction of Sn^4+^ to Sn^2+^ by additional SnC_2_O_4_. b) Schematic diagram of Sn^4+^ reduction by oxalate. Sn 3d_5/2_ XPS spectra of control and 4% SnC_2_O_4_ treated perovskite films in c) fresh; d) after light/ambient air exposure for 30 min.

Mixed Sn‐Pb narrow‐bandgap perovskites with a component of FA_0.5_MA_0.5_Pb_0.5_Sn_0.5_I_0.47_Br_0.03_ with or without SnC_2_O_4_ addition are prepared as light absorbers. The molar concentration of SnC_2_O_4_ is 2% and 4% of the FA_0.5_MA_0.5_Pb_0.5_Sn_0.5_I_0.47_Br_0.03_. Since the 4% SnC_2_O_4_ added solution is saturated, larger concentrations are not studied. (henceforth, 2% and 4% SnC_2_O_4_ are marked for the samples). The reducibility of SnC_2_O_4_ in precursor solution is first investigated. **Figure** **1**a shows photos of the control and SnC_2_O_4_ perovskite precursor solutions at different stages. Both prepared perovskite solutions exhibit a translucent pale yellow. After being exposed to ambient air for 1 h, the solutions change from pale yellow to reddish brown, indicating severe oxidation of the solution and the formation of a large amount of Sn^4+^ in the solutions, which is achieved by Equation (1).^[^ ^8^, ^16^ ^]^ It is interesting to note that the solution with 4% SnC_2_O_4_ also turns red because large amounts of Sn^4+^ are produced under exposure to air. 4% SnC_2_O_4_ does not guarantee a complete reduction against the ambient oxidation rate. Subsequently, an excess of SnC_2_O_4_ is added to the solution, which gradually returns to pale yellow color. A more detailed restoration process is shown in Figure S1 (Supporting Information). After 20 h of reduction, Sn^4+^ ions are effectively reduced due to the presence of chemically reduced C_2_O_4_^2−^ through Equation (2), and the solution is restored to its original pale yellow. The schematic diagram of C_2_O_4_^2−^ reduction of Sn^4+^ is shown in the illustration of Figure 1b. Moreover, the reaction product is CO_2_ which escapes from the solution without any pollution.^[^ ^17^ ^]^

(1) $$2Sn^{2+}+O_{2}\to 2Sn^{4+}+2O^{2-}$$

(2) $${\left(C_{2}O_{4}\right)}^{2-}+Sn^{4+}\to Sn^{2+}+2CO_{2}$$

The reduction of SnC_2_O_4_ toward Sn^4+^ is also confirmed by the X‐ray photoelectron spectroscopy (XPS) measurements in the absence of light in air shown in Figure 1c. The XPS spectrum of Sn 3d_5/2_ can be divided into two sub‐peaks, with low energy peaks (486.5 eV) attributed to Sn^2+^ and high energy peaks (487.2 eV) to Sn^4+^.^[^ ^18^ ^]^ Compared to the control film, the area ratio of Sn^4+^ in 4% SnC_2_O_4_ film is decreased from 28.7% to 10.4%, more Sn^2+^ is observed in the 4% SnC_2_O_4_ film, suggesting that after adding SnC_2_O_4_, the content of Sn^2+^ increases by extra Sn^2+^ addition or reducing Sn^4+^.

Under Light/oxygen conditions, superoxide is formed on the perovskite surface by the interaction of a neutral oxygen molecule and a photo‐excited electron.^[^ ^19^ ^]^ Therefore, when the perovskites are exposed to light and oxygen, photo‐excited electrons are formed in the perovskite and readily transfer from the perovskite surface to oxygen to generate superoxide.^[^ ^20^ ^]^ Superoxide can cause the oxidation of Sn^2+^ to Sn^4+^ and the escape of iodine, leading to the decomposition of perovskite structure and severe degradation of performance.^[^ ^21^ ^]^ The degradation of perovskites upon irradiation to yield Pb^0^.^[^ ^22^ ^]^ To investigate the role of SnC_2_O_4_ on the stability of perovskite, the Pb 4f XPS spectra before and after degradation are presented. Fresh samples are taken directly after spin‐coating the perovskite, while light/ambient air samples are exposed to 1 sunlight in ambient air for 30 min to simulate perovskite degradation. As shown in Figure S2 (Supporting Information), for the control film, the peaks with a binding energy of 142.9 and 138 eV are attributed to the 4f_5/2_ and 4f_7/2_ of Pb^2+^.^[^ ^23^ ^]^ After irradiation under light/ambient air conditions for 30 min, the control sample shows a peak of Pb^0^ at 135.8 eV, demonstrating degradation of perovskite. In contrast, the sample with 4% SnC_2_O_4_ did not demonstrate the Pb^0^ peak, indicating that SnC_2_O_4_ effectively inhibited the degradation of the perovskite film. This is also evidenced by the changes in the UV–Vis absorption spectra (UV–vis) in Figure S3 (Supporting Information). For the control sample, the decrease in absorption from 500 to 900 nm after 30 min under light/oxygen conditions indicates the decline of the perovskite. While the absorption of the SnC_2_O_4_ sample remains almost the same, indicating better stability of the SnC_2_O_4_ modified film. In addition, the 4% SnC_2_O_4_ film shows less Sn^4+^ after light exposure in an aerobic environment (Figure 1d), suggesting that SnC_2_O_4_ plays a key role in inhibiting Sn^2+^ oxidation. As a result, due to the reductive C_2_O_4_^2−^, more Sn^2+^ is retained, which helps to optimize perovskite films with fewer defects and higher stability.

**Figure 2:**
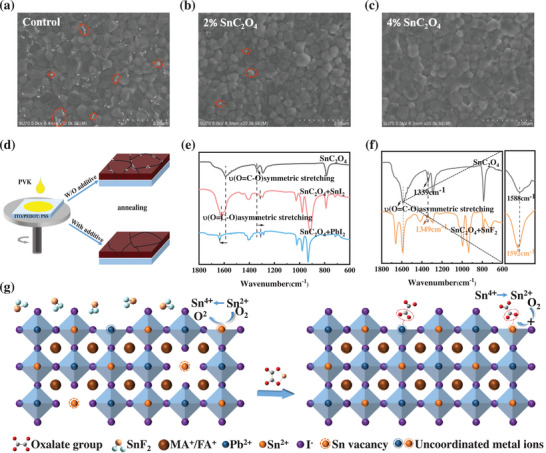
a–c) Top‐view SEM images of thin films with distinct SnC_2_O_4_ doping rates. d) Schematic illustration of the preparation of the perovskite film and the morphologies of the perovskite films with and without additive. e) FTIR spectra of pure SnC_2_O_4_, SnC_2_O_4_ +SnI_2_ complex, and SnC_2_O_4_ +PbI_2_ complex in DMF and DMSO. f) FTIR spectra of pure SnC_2_O_4_, SnC_2_O_4_ +SnF_2_ complex in DMF and DMSO. g) Schematic illustrating the functions of SnC_2_O_4_.

To elucidate the effect of SnC_2_O_4_ on the perovskite crystallization, the crystallinity and morphology of the films are explored. **Figure** **2**a–c shows the top‐view scanning electron microscopy (SEM) images of thin films with different SnC_2_O_4_ doping amounts (For more detailed information in Figure S4, Supporting Information). After the addition of different concentrations of SnC_2_O_4_, the grain sizes slightly increased, and the film surface became smoother. It is worth noting that “bright spots” are present at the grain boundaries of the control film. X‐ray diffraction (XRD) and energy dispersive X‐ray (EDX) spectroscopy tests are performed to characterize such aggregates. As shown in Figure S5 (Supporting Information), the diffraction peak intensities are enhanced with increasing additive concentration, indicating the SnC_2_O_4_ perovskite films exhibit enhanced crystallinity.^[^ ^24^ ^]^ It is noteworthy that a characteristic peak of PbI_2_ appears at 12.77° in the control film, which is attributed to the unreacted precursor material or the degraded perovskite.^[^ ^4^, ^25^ ^]^ This severely hinders the interfacial carrier transport between the active and charge‐transport layers and increases hysteresis.^[^ ^26^ ^]^ For perovskite modified with 4% SnC_2_O_4_, the peak intensity of PbI_2_ disappeared, suggesting that the addition of SnC_2_O_4_ interacted with PbI_2_ to eliminate PbI_2_ phase formation. From EDX elements analysis in Figure S6 (Supporting Information), the content of elements (F/Sn/Pb/I) in the “bright spot” increased compared to the blank area, which proves the “bright spots” may be amorphous compounds of SnF_2_ and PbI_2_.^[^ ^12^, ^27^ ^]^ After adding 2% additives, the “bright spots” are reduced, while “bright spots” completely disappeared at 4% SnC_2_O_4_ content (Figure S4, Supporting Information), which may be due to the full removal of SnF_2_ and PbI_2_ by sufficient SnC_2_O_4_. As shown in Figure S7 (Supporting Information), the solution changed from turbid with powder precipitation to clear with flaky precipitation after added SnF_2_, proving the complexation of SnF_2_ with SnC_2_O_4_. Figure 2d shows the procedure and effects of preparing perovskite films with or without additives. In short, the addition of SnC_2_O_4_ eliminates residues and makes the film surface smoother, which can be beneficial for carrier transport and improve device performance.

Subsequently, Fourier transform infrared (FTIR) spectroscopy is used to investigate the interaction of SnC_2_O_4_ and perovskite (Figure S8, Supporting Information). The C═N vibrational band at 1712 cm^−1^ in perovskite membranes is assigned to the FA^+^ cation. The C‐N vibration at 1612 cm^−1^ originated from the MA^+^ cation. The peak at 792 cm^−1^ could be attributed to the O─C═O deformation in the oxalate, suggesting that SnC_2_O_4_ is successfully doped into perovskite.^[^ ^28^ ^]^ Besides, the shift of the O─C═O deformation peak towards higher wave numbers indicates an interaction between SnC_2_O_4_ and perovskite. More importantly, both symmetric and antisymmetric stretching (O═C─O) of SnC_2_O_4_ shift in the mixed film of SnC_2_O_4_ and PbI_2_/SnI_2_ (Figure 2e), indicating the interaction between oxalate and Sn^2+^/Pb^2+^ ions. Moreover, as shown in Figure 2f, both the symmetric and asymmetric scaling (O═C─O) shifted in the mixed film of SnC_2_O_4_ and SnF_2_, which shows the interaction of SnC_2_O_4_ and SnF_2_. The disappearance of the “bright spots” by adding SnC_2_O_4_ might be ascribed to the chemical coordination between PbI_2_, SnF_2_, and SnC_2_O_4_. As a Lewis base, the C═O of the oxalate can form coordination bonds with uncoordinated Sn^2+^/Pb^2+^, which can passivate defects by Lewis acid‐base interaction and thus improve the performance and stability of the device.^[^ ^29^ ^]^

To further investigate the chemical change and potential bonding reaction in perovskite, XPS is carried out. We observed that the binding energy of metal cations and halide ions shifted towards lower energies compared to the control (Figure S9, Supporting Information), where the Sn 3d_5/2_ and Sn 3d_3/2_ binding energies shifted from 486.58 and 495.0 to 486.51 eV and 494.95 eV, respectively. F 1s decreases from 684.35 to 684.25 eV. The characteristic peaks of Pb and I elements are also slightly shifted, indicating the chemical environment around the atoms changes due to the effect of SnC_2_O_4_. The peak shift is attributed to a change in the chemical environment due to the interaction of the high electron density group C═O in C_2_O_4_^2−^ with Sn^2+^/Pb^2+^. The schematic diagram of the role of SnC_2_O_4_ in Sn‐Pb perovskite film is shown in Figure 2g. First, SnC_2_O_4_ fills V_Sn_ and chelates with uncoordinated metal ions, resulting in a film with a low defect density of states. Second, excess PbI_2_ and SnF_2_ on the surface of the film are eliminated to obtain a smooth film for carrier transport. Furthermore, Sn^4+^ is reduced to Sn^2+^ by reductive C_2_O_4_^2−^, which inhibits the generation of V_Sn_ and improves the device's performance.

**Figure 3:**
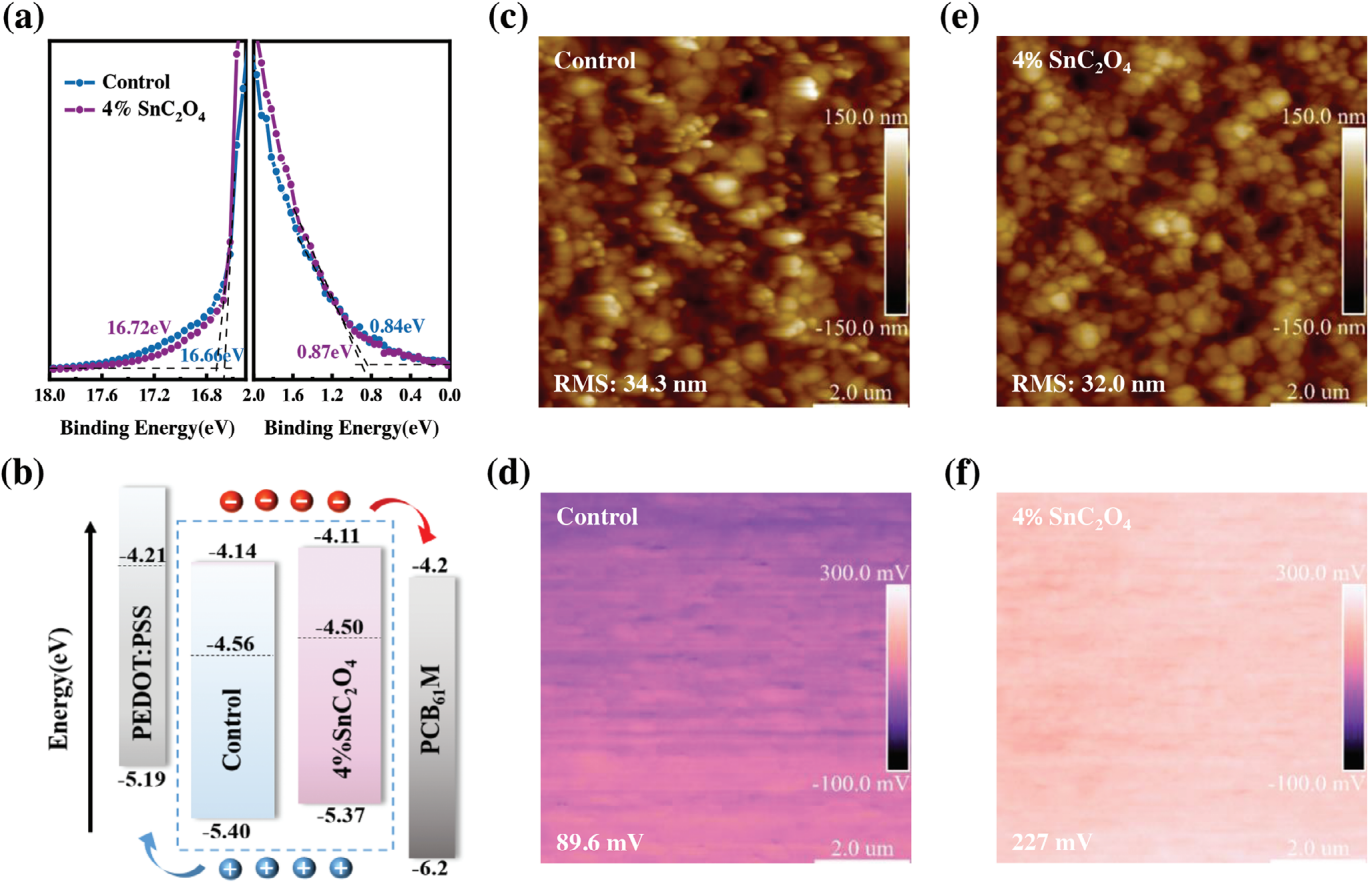
a) UPS spectra of secondary electron cutoff (E_cutoff_) and onset (E_onset_) energy of the control and 4% SnC_2_O_4_ film. b) Energy level diagram constructed from UPS results. AFM morphology of c) control and (e) 4% SnC_2_O_4_ treated perovskite films. KPFM results of d) control and f) 4% SnC_2_O_4_ treated perovskite films.

The oxidation of Sn^2+^ to Sn^4+^ in Sn‐Pb perovskite leads to p‐type self‐doping characteristics that are detrimental to the device's performance.^[^ ^5b^ ^]^ The energy band structure modulation of SnC_2_O_4_‐modified perovskite films is investigated by ultraviolet photoelectron spectroscopy (UPS). **Figure** **3**a shows the secondary electron cutoff (E_cutoff_) and onset (E_onset_) energies of the obtained control and 4% SnC_2_O_4_ films, respectively. Detailed parameters of the energy band structure, such as the positions of the Fermi energy levels (E_F_), conduction band (CB), and valence band (VB), are determined in conjunction with the bandgap (Figure S10, Supporting Information). The corresponding parameters are summarized in Table S1 (supporting information). The values of VB for control and 4% SnC_2_O_4_ film are −5.40 and −5.37 eV, respectively, according to their bandgap (Eg = 1.26 eV), the values of CB are estimated to be −4.14 and −4.11 eV, respectively. As Figure 3b shows, the SnC_2_O_4_ modification could promote the E_F_ and CB of perovskite film. The elevated E_F_ and CB are helpful for reducing electron transfer barrier and interface nonradiative recombination loss; thus, improving the charge transport and extraction ability of the corresponding device.^[^ ^30^ ^]^ The VB energy band edge of the 4% SnC_2_O_4_ film is closer to the energy band of PEDOT: PSS, which means a better energetic match between the perovskite and hole transport layer. The overall energy level of the film modified with SnC_2_O_4_ shifts upwards, which facilitates better energy level matching, to reduce the interface energy loss and promote the separation and transfer of photo carriers in the device.

Atomic force microscopy (AFM) and kelvin probe force microscopy (KPFM) measurements are performed to analyze Sn‐Pb mixed perovskite films with and without 4% SnC_2_O_4_. As shown in Figures 3c,e, the film introduced with additives exhibits a relatively flat and smooth morphology, which is reflected by the results of the SEM image in Figure 2a–c. The root‐mean‐square roughness (Rq) values of control and 4% SnC_2_O_4_ films are estimated to be 34.3 and 32.0 nm, respectively, suggesting that the introduction of SnC_2_O_4_ is beneficial for uniform perovskite films, which is beneficial for carrier transport.^[^ ^31^ ^]^ Additionally, contacting potential difference (CPD) morphology images obtained from KPFM demonstrated that surface electrical properties of the perovskite have been changed by the SnC_2_O_4_, with the average CPD increasing from 89.6 to 227 mV (Figure 3d,f). A larger CPD value means a smaller work function in the 4% SnC_2_O_4_ film, agreeing well with the UPS results, which is conducive to the extraction of charge carriers. Then we randomly obtain the surface potential distributions of control and 4% SnC_2_O_4_ films on KPFM plots, the 4% SnC_2_O_4_ films show a more uniform potential distribution (Figure S11, Supporting Information). The uniform distribution of surface contact potential is beneficial for effective carrier extraction and helps suppress non‐radiative recombination, thus enabling the manufactured devices to have superior performance.^[^ ^32^ ^]^

**Figure 4:**
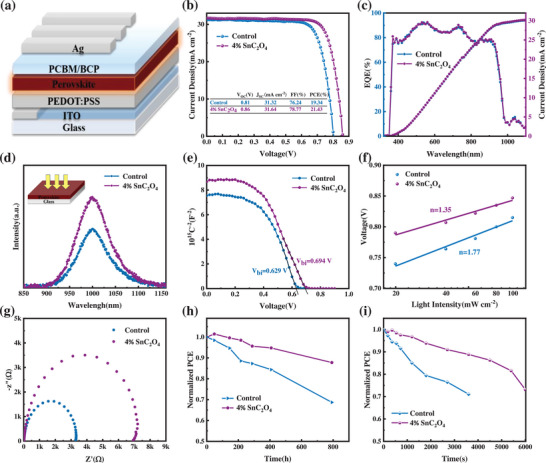
a) Device structure. b) The *J‐V* curves of the control and 4% SnC_2_O_4_ treated device. c) EQE plots and derived integrated *J_SC_* of the control and 4% SnC_2_O_4_ treated device. d) steady‐state photoluminescence (PL) spectra. e) Mott‐Schottky analysis at 1 kHz of the Sn‐Pb films of the control and 4% SnC_2_O_4_ treated device. f) *V_OC_* dependence on the light intensity of different Sn‐Pb PSCs. The results are fitted with a linear function. g) Nyquist plots of the control and 4% SnC_2_O_4_ treated device. h) Shelf stability of control and 4% SnC_2_O_4_ devices in glove box without encapsulation. i) Device stability under aerobic conditions with continuous light irradiation.

To gain insight into the effect of the SnC_2_O_4_ modification on the photovoltaic performance, p‐i‐n planar PSCs with a configuration of ITO/PEDOT: PSS/perovskite/PCBM/BCP/Ag is fabricated and the schematic structure is shown in **Figure** **4**a. Based on the *J‐V* curve (Figure S12, Supporting Information) and the parameters summarized in Table S2 (Supporting Information), it is concluded that a 4% molar ratio of SnC_2_O_4_ additive shows the best device performance, while an insufficient amount of SnC_2_O_4_ additive cannot fully remove the residue on the grain boundary, thus resulting in limited improvement in device performance. The photovoltaic parameter distribution of 20 cells for each condition is summarized in Figure S13 (Supporting Information), confirming the good reproducibility of the devices. Figure 4b shows the *J‐V* curve of the champion PSC based on control and 4% SnC_2_O_4_ film. The 4% SnC_2_O_4_ device exhibits overall enhancement on open circuit voltage (*V_OC_*) and fill factor (*FF*), resulting in a PCE of up to 21.43% (reverse scanning, Figure S14, Supporting Information), much higher than the 19.34% of the control device. The detailed photovoltaic parameters are shown in the illustration of Figure 4b. For devices without and with 4% SnC_2_O_4_ additives, the reliability of short‐circuit current density (*J_SC_*) obtained from *J‐V* curves is verified within the error range by measuring the external quantum efficiency (EQE) (Figure 4c).

The significant performance improvement of the device mainly stems from the significant enhancement of *V_OC_* and *FF*, which is associated with a reduction in defect density. Thereby, steady‐state photoluminescence (PL), space charge limited current (SCLC), and dark current measurements are conducted to investigate the effect of SnC_2_O_4_ modification on defect passivation and carrier recombination. As shown in Figure 4d, 4% SnC_2_O_4_ film exhibits a stronger PL emission peak compared to the control film, indicating that non‐radiative recombination within the 4% SnC_2_O_4_ film is significantly suppressed, reducing defect density. Devices with a dual‐hole transport layer of PEDOT: PSS and Spiro‐OMeTAD are used to further test defect densities in control and 4% SnC_2_O_4_ films. The dark *J‐V* curves can be divided into three regions according to the *J*∝*V^n^* relation. When *n *= 1, 2, and 3, the figure showed the ohmic region, trap filling limit (TFL) region and space charge limited current region, respectively. The *V_TFL_* of devices with or without 4% SnC_2_O_4_ is 0.289 and 0.398 V, respectively (Figure S15, Supporting Information). Correspondingly, the trap state density of the 4% SnC_2_O_4_ device decreased from 2.14 × 10^15^ to 1.55 × 10^15^ cm^−3^. It is evidenced that devices based on 4% SnC_2_O_4_ exhibit lower trap density, indicating that non‐radiative recombination in PSCs can be significantly suppressed.^[^ ^33^ ^]^ The significant decrease in defect density of SnC_2_O_4_ films is related to the reduction of V_Sn_, and uncoordinated metal ions, as mentioned earlier.

The Mott‐Schottky measurements are conducted to estimate the built‐in potential (*V_bi_*) of the Sn‐Pb mixed perovskite device before and after adding SnC_2_O_4_ to elucidate the increase in *V_OC_* and *FF* (Figure 4e). It is evidenced that the modified device with 4% SnC_2_O_4_ exhibits a higher *V_bi_* of 0.694 V compared to the control device with a *V_bi_* of 0.629 V. The increased *V_bi_* can be attributed to the reduction of charge defects at the perovskite surface and the formation of a more favorable electronic structure at the perovskite/PCBM interface. A higher *V_bi_* is conducive to the separation and extraction of charge carriers, thus contributing to higher *FF* and *V_OC_* of the devices. The ideality factor (*n*) is another measure of merit that could be used to estimate the charge extraction and recombination processes of devices.^[^ ^34^ ^]^ Thereafter, we tested the *J‐V* characteristics of the devices at light intensities from 0.2 to 1 sun. Figure 4f illustrates the relationship between *V_OC_* and the logarithmic light intensity. It can be seen that the 4% SnC_2_O_4_ device has an *n* value of 1.35, whereas the control device had an *n* value of 1.77, elucidating that the trap‐assisted charge carrier recombination is pronouncedly suppressed after SnC_2_O_4_ modification, which is conducive to improve *V_OC_* and *FF*. Besides, a more ideal *α* value (0.92) for the 4% SnC_2_O_4_ device (Figure S16, Supporting Information) suggests the formation of high‐quality perovskite film with better energy level alignment between perovskite and charge transport layer, thus facilitating charge extraction and collection.

Then, the dark current is recorded by measuring the *J‐V* curve of solar cells under dark conditions. As shown in Figure S17 (Supporting Information), the device modified with 4% SnC_2_O_4_ has a much lower dark current under both positive and negative biases compared with the control device, indicating a much lower leakage current and non‐radiative recombination in the modified device. In addition, the steep slope of the dark *J‐V* curves for the 4% SnC_2_O_4_ device in the high forward bias voltage region indicates low series resistance and efficient charge transport properties, which contributes to the improvement of the *V_OC_* and *FF*. In addition, Figure 4g shows the electrochemical impedance spectroscopy (EIS) diagrams of the 4% SnC_2_O_4_ device have a larger semicircle corresponding to a larger recombination resistance (Rrec), indicating a lower charge recombination rate compared to the control device.^[^ ^33^ ^]^ Moreover, the transient photo‐voltage attenuation (TPV) in Figure S18 (Supporting Information) shows an extension of the decay time of the 4% SnC_2_O_4_ device due to the reduction of recombination in SnC_2_O_4_. Thus, the carrier lifetimes are greatly extended. The transient photocurrent attenuation (TPC) of PSCs with 4% SnC_2_O_4_ (Figure S19, Supporting Information) decreases from 4.97 µs to 3.52 µs, which shows 4% SnC_2_O_4_ devices have better carrier transport capabilities. That may be the result of a more matched energy level structure between perovskite and hole transport layer with 4% SnC_2_O_4_ added to promote charge extraction transport. Therefore, the results again indicated that modification with 4% SnC_2_O_4_ can effectively promote charge transfer and reduce carrier recombination at the buried interface of the device.

At last, we monitored the long‐term storage stability and light stability of narrow‐bandgap Sn‐Pb PSCs incorporated without or with SnC_2_O_4_ doping. As exhibited in Figure 4h, the unencapsulated 4% SnC_2_O_4_‐doped Sn‐Pb PSC retained 78% of its initial efficiency after storage in a nitrogen (N_2_) filled glovebox for ≈1000 h at room temperature. By contrast, the efficiency of the control device maintained only 68% of its original value after being for ≈800 h under the same aging conditions. Moreover, the light stability of the device is measured under light/oxygen conditions (Figure 4i). The device with 4% SnC_2_O_4_ shows better photostability than the control device. Therefore, in addition to improving device efficiency, SnC_2_O_4_ doping could also enhance solar cell stability, which could be mainly attributed to the improved crystalline quality, reduced trap density, and suppressed recombination of the SnC_2_O_4_‐doped Sn‐Pb perovskite films.

## 3 Conclusion

In summary, we have shown that the addition of SnC_2_O_4_ can effectively modify narrow bandgap Sn‐Pb perovskite solar cells. The use of SnC_2_O_4_ as a Sn^2+^ compensating agent, reductive agent toward Sn^4+^, and a surface coordinator for passivation of surface defects, greatly enhances the quality of Sn‐Pb perovskite film and energy band alignment of the interface. Our best‐performing narrow bandgap PSC achieves a championship PCE of 21.43%, and the long‐term and light stability of the SnC_2_O_4_‐modified device is greatly improved.

## Conflict of Interest

The authors declare no conflict of interest.

## Supporting information

Supporting Information

## Data Availability

The data that support the findings of this study are available from the corresponding author upon reasonable request.
