# Supplementary material for: Reductive Sn2+ Compensator for Efficient and Stable Sn‐Pb Mixed Perovskite Solar Cells
Source: Adv Sci (Weinh). 2024 Apr 18;11(25):2400962. doi: 10.1002/advs.202400962 (PMC11220707; doi:10.1002/advs.202400962)
Supplement: Supplementary file 1 — Supporting Information [file ADVS-11-2400962-s001.pdf]

ADVANCED SCIENCE
Open Access

Supporting Information

for *Adv. Sci.*, DOI 10.1002/advs.202400962

Reductive Sn^{2+} Compensator for Efficient and Stable Sn-Pb Mixed Perovskite Solar Cells

*Qiuxiang Wang, Jiaxing Xiong, Yanjun Xing, Xinlei Gan, Wendong Zhu, Rong Xuan, Xiaohui Liu, Like Huang, Yuejin Zhu and Jing Zhang**

Reductive Sn^{2+} Compensator for Efficient and Stable

Sn-Pb Mixed Perovskite Solar Cells

*Qiuxiang Wang, Jiaxing Xiong, Yanjun Xing, Xinlei Gan, Wendong Zhu, Rong Xuan, Xiaohui Liu, Like Huang, Yuejin Zhu, Jing Zhang**

Experimental Section

Materials: Indium doped tin oxide (ITO) conductive glass with a square resistance of 10 Ω/sq and tin iodide (SnI_2, 99.999%) are purchased from Advanced Electron Technology Co., Ltd. Lead iodide (PbI_2, 99.999%), lead bromide ($PbBr_2$, 99.99%), methylammonium bromide (MABr, 99.5%), methylammonium iodide (MAI, 99.5%), formamidine hydroiodide (FAI, 99.5%), [6,6]-phenyl-C61-butyric acid methyl ester ($PC_{61}BM$, 99.9%), Poly(3,4-ethylenedioxythiophene): polystyrene sulfonate (PEDOT: PSS, Al 4083), Fullerene (C60, 99%), and 2,9- dimethyl-4,7-diphenyl-1,10-phenanthroline (BCP, 99%) are purchased from Xi'an Yuri Solar Co., Ltd. The tin fluoride (SnF_2, 99%), and lead thiocyanate ($Pb(SCN)_2$, 99.5%), Sn powder (99.99%) are purchased from Aladdin. Tin(II) oxalate (SnC_2O_4, 99%) is purchased from Solarbio. N, N-Dimethylformamide (DMF, 99.8%, super dry), and dimethyl sulfoxide (DMSO, 99.7%, super dry) are obtained from J&K Scientific Ltd. Chlorobenzene (CB, 99.8%) are purchased from Aladdin. Unless otherwise stated, all chemicals and materials are purchased and used on receipt.

$FA_{0.5}MA_{0.5}Sn_{0.5}Pb_{0.5}I_{0.47}Br_{0.03}$ Film preparation: The $FASnI_3$ solution is prepared by dissolving of SnI_2 (0.3725g), FAI (0.172g), and SnC_2O_4 (0.0082g) with 5 mol% of SnF_2 in DMF/DMSO (2:1). The $MAPbI_3$ precursor solution is prepared by dissolving PbI_2 (0.461g) and MAI (0.159g) with 5 mol% of $Pb(SCN)_2$ in DMF/DMSO (8:1). The $MAPbBr_3$ precursor solution is prepared by dissolving MABr (0.112g) and $PbBr_2$ (0.367g) in DMF/DMSO (8:1). Excess Sn powder is added into the $FASnI_3$ solution precursor during the dissolution process, and then filter the solution by a 0.22 μm

PVDF-filter. The $FA_{0.5}MA_{0.5}Sn_{0.5}Pb_{0.5}I_{0.47}Br_{0.03}$ precursor solution is then formed by mixing stoichiometric amounts of $FASnI_3$, $MAPbI_3$and $MAPbBr_3$ perovskite precursor.

Device fabrication: Indium doped tin oxide (ITO) glass substrate is ultrasonically cleaned with deionized water and alcohol, respectively, for 15 minutes at a time. PEDOT: PSS aqueous solution films are coated on the cleaned ITO substrate at 4000 rpm for the 30s and then dried at 150 °C for 15 min in ambient air. The substrates are then transferred into a N_2-filled glovebox for the deposition of perovskite films. The 1.6M $FA_{0.5}MA_{0.5}Sn_{0.5}Pb_{0.5}I_{0.47}Br_{0.03}$ solution is spin-coated onto the PEDOT: PSS substrate at 4000 rpm for 30 s, and CB (180 μl) is dropped onto the substrate at 20 s and then dried at 100 ℃ for 5 min. Subsequently, the PCBM (40μl, 10 mg mL^{-1}) solution in chlorobenzene is deposited on perovskite film at 2500 rpm for 30 s and then dried at 60 ℃ for 10 min. Then the samples are put into a high vacuum chamber for the following deposition of functional layers. 23 nm C_{60} and 7 nm BCP are sequentially evaporated onto the perovskite. 70 nm Ag is finally deposited.

Characterizations:

Atomic force microscopy (AFM) is conducted on Veeco (America) and Kelvin probe force microscopy (KPFM) is used to detect the contact potential to reveal the fermi level and ion migration properties of the film under the bias applied normally to the film. The surface morphology of the films and the cross-section morphology of the devices are tested by a Hitachi SU-70 scanning electron microscope (SEM). X-ray diffraction (XRD) patterns of films are tested by a Bruker D8 advanced instrument and using Cu Kα as radiation (λ=6.162Å) at a scan rate of 4 ° min^{-1} and diffraction angle range from 10 ° to 40 °. The absorption spectra of the film on the glass substrate are measured by a UV-visible spectrophotometer (Agilent, USA).

FTIR spectroscopy is taken with an FTIR spectrometer instrument (Thermo, Nicolet 6700). The binding energies of the elements in PVK are tested by X-ray photoelectron spectroscopy (XPS, Shimadzu, Japan) using Al Kα radiation. Au is used to calibrate the energy state of the spectroscopy before measurement. The ultraviolet photoelectron

spectroscopy (UPS) pattern is detected by the Axis Ultra DLD, and using the He I (21.22 eV) emission line. The steady-state fluorescence (PL) spectra of the films are measured based on the glass by a fluorescence spectrophotometer (Agilent, USA), with an excitation wavelength of 532 nm.

The SCLC measurements and dark J-V curves are measured by the Keithley 4200 under dark conditions. The J-V characteristic curves are tested by the Keithley 4200 meter and the sunlight simulator (Newport, 91192A, AM 1.5, 1 sun) whose light intensity is calibrated by a standard silicon solar cell. The solar cells are masked with a black aperture to define the active area of 0.08 cm^2. The light intensity is calibrated to AM 1.5G (100 $mWcm^{-2}$) by using a reference Si solar cell. The external quantum efficiency (EQE) spectra are recorded with the Newport EQE system in which the light intensity at every wavelength is calibrated with a Si detector before measurement and the wavelength range from 300 to 1100 nm. The transient photo-current (TPC) decay and transient photo-voltage (TPV) decay are recorded by an electrochemical workstation (Zahner, Germany) with a white light LED supplied 80 $mWcm^{-2}$ light intensity to excite the perovskite solar cells. Electrochemical impedance spectroscopy (EIS) is performed in the frequency range from 10 Hz to 1 MHz by an electrochemical workstation in dark conditions with a bias of 0.6 V.

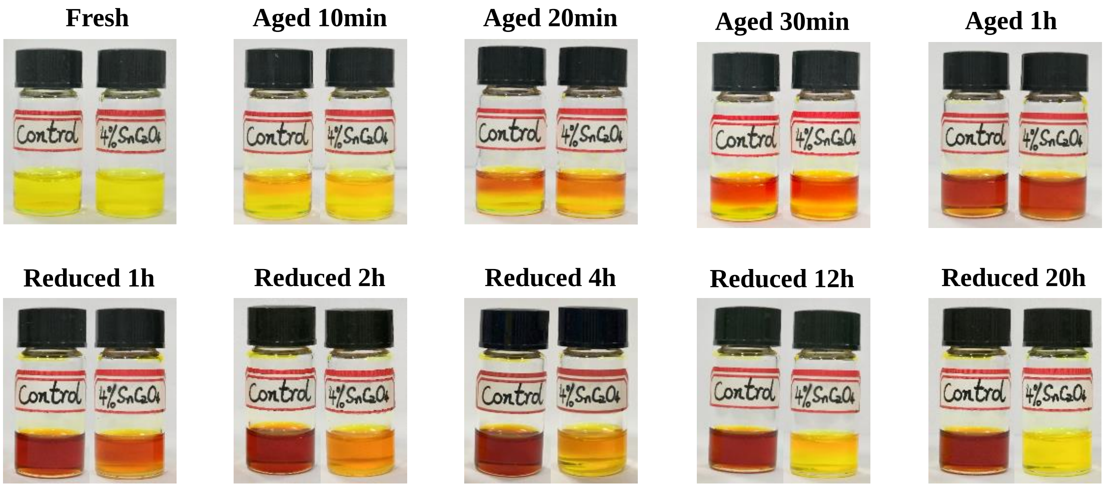

Figure S1 Digital photos of mixed Pb-Sn perovskite precursor solutions, showing the oxidation of Sn^{2+} (yellow in solution) to Sn^{4+} (red in solution) and the reduction of Sn^{4+} to Sn^{2+} by SnC_2O_4. During exposure to air, the vials were kept open (uncapped).

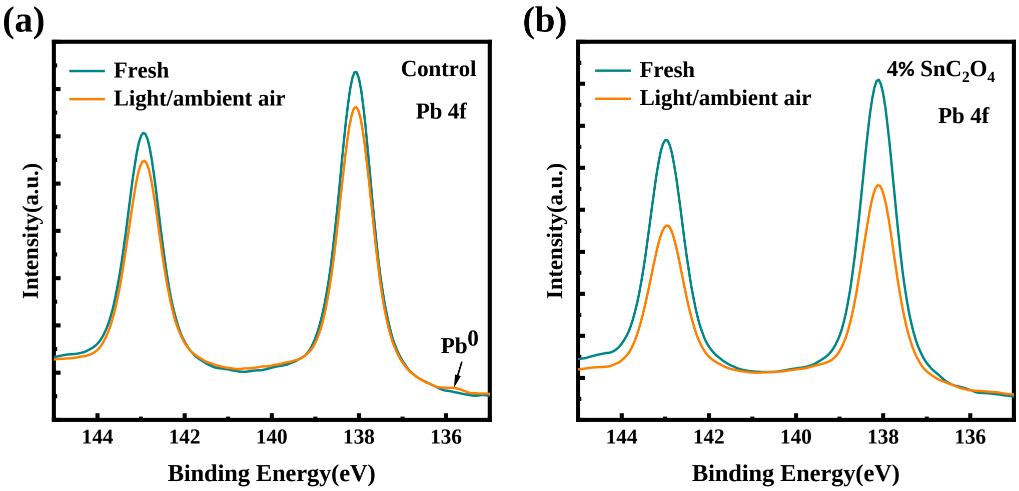

Figure S2. XPS spectra of Pb 4f of perovskite films of fresh or light/ambient air (a) control, (b) 4% SnC_2O_4.

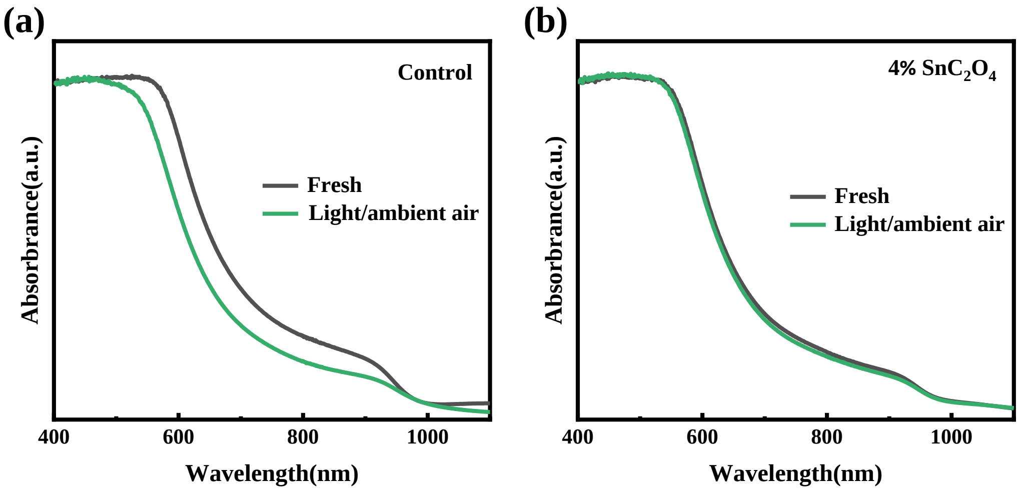

Figure S3. UV-vis absorption of perovskite films of fresh or light/ambient air. (a) control, (b) 4% SnC_2O_4.

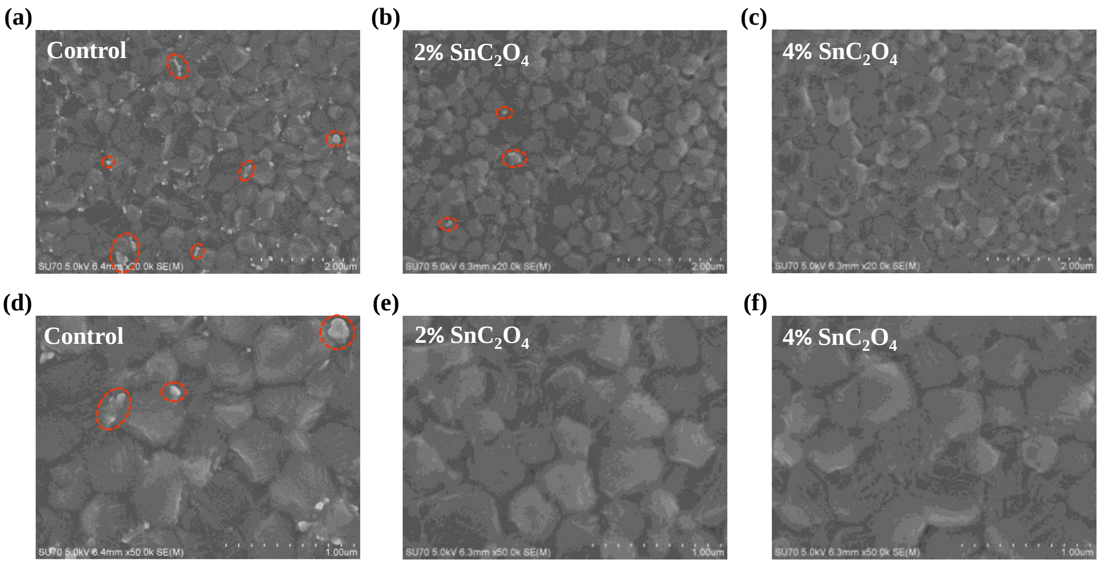

Figure S4. SEM images of thin films without or with distinct SnC_2O_4 doping rates.

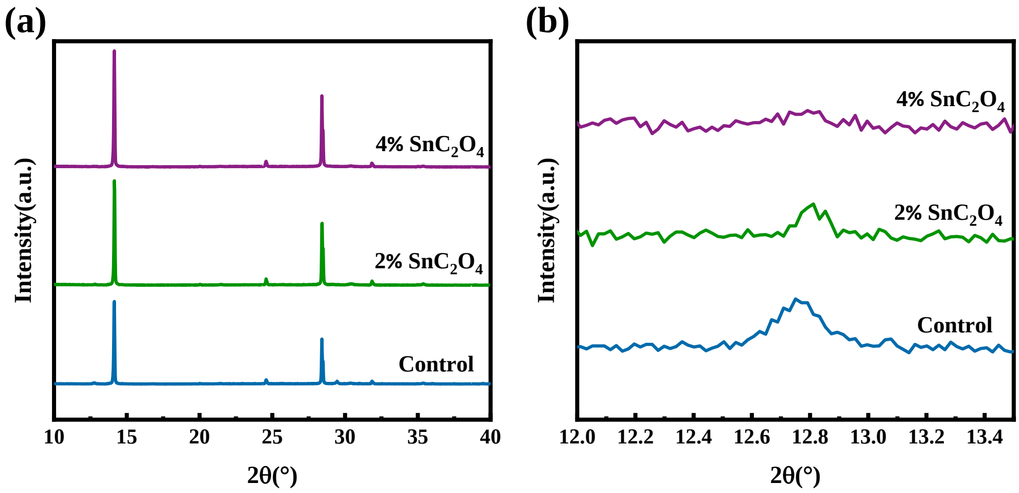

Figure S5. XRD spectra of perovskite film without or with distinct SnC_2O_4 doping rates.

(a) (b)

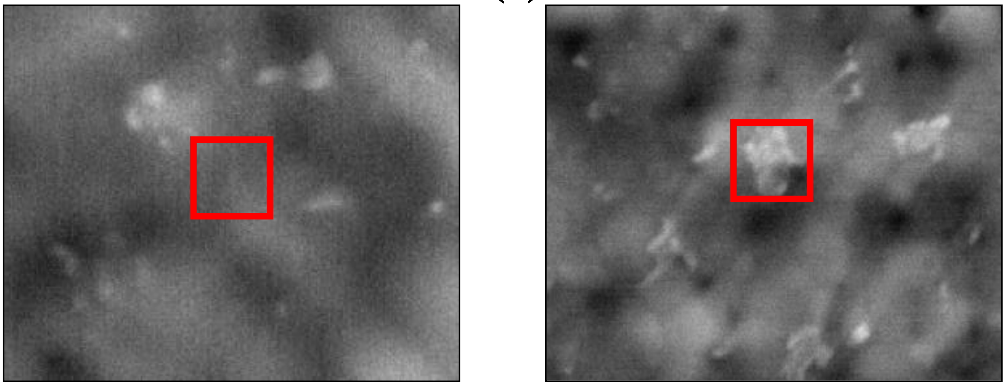

(c)

Element	Wt%	At%
CK	02.47	17.25
OK	03.71	19.45
FK	00.74	03.27
PbM	15.31	06.20
InL	26.79	19.59
SnL	10.97	07.76
IL	40.02	26.47
Matrix	Correction	ZAF

(d)

Element	Wt%	At%
CK	02.53	17.40
OK	03.71	19.14
FK	01.25	05.45
PbM	16.65	06.63
InL	22.93	16.47
SnL	11.39	07.92
IL	41.52	26.99
Matrix	Correction	ZAF

Figure S6. EDS spectra of perovskite film without SnC_2O_4 addition.

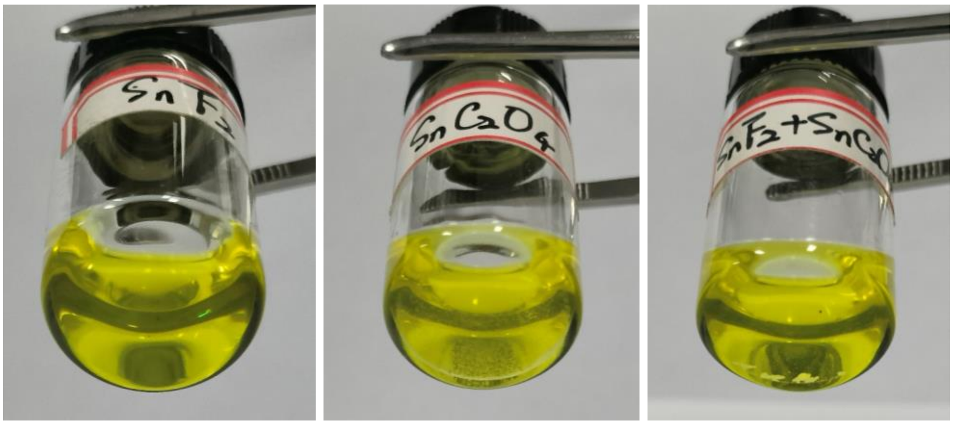

Figure S7. Schematic diagram of SnF_2 complexation with SnC_2O_4.

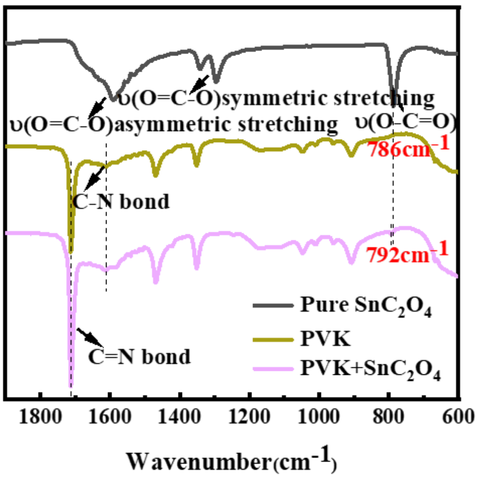

Figure S8. FTIR spectroscopy for SnC_2O_4, control and SnC_2O_4-doped films.

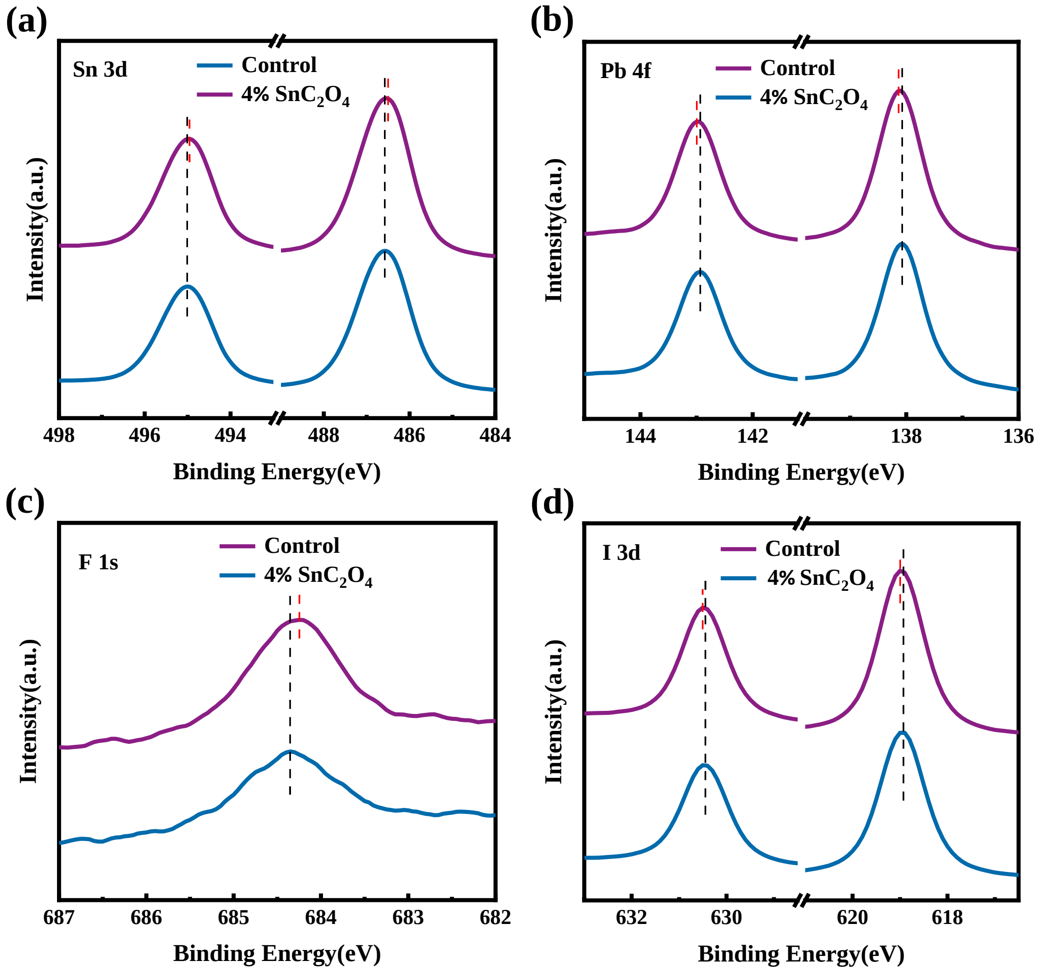

Figure S9. X-ray photoelectron spectroscopy of (a) Sn 3d, (b) Pb 4f, (c) F 1s, (d) I 3d.

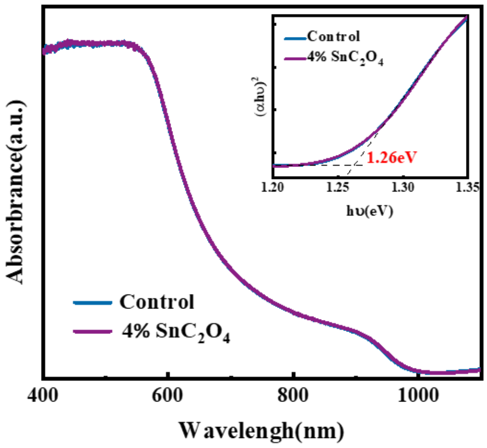

Figure S10. UV-vis absorption of control and 4% SnC_2O_4 films.

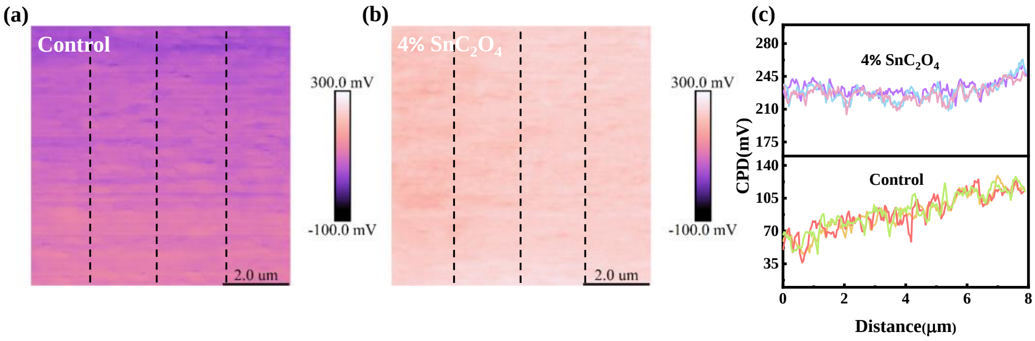

Figure S11. Surface potential mapping at the same topography area of (a) control (b) 4% SnC_2O_4, and (c) their contact potential difference (CPD) line profiles.

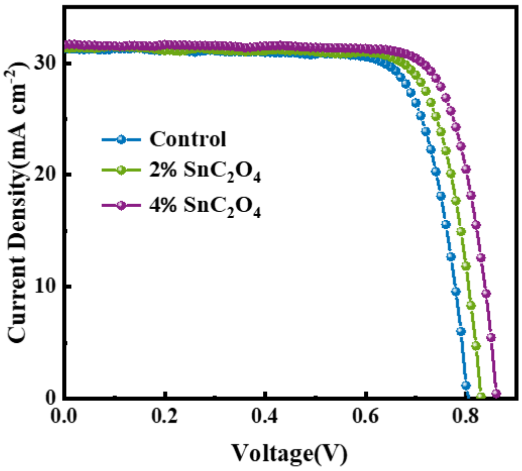

Figure S12. The J–V curves of the control, 2% SnC_2O_4 and 4% SnC_2O_4 treated device.

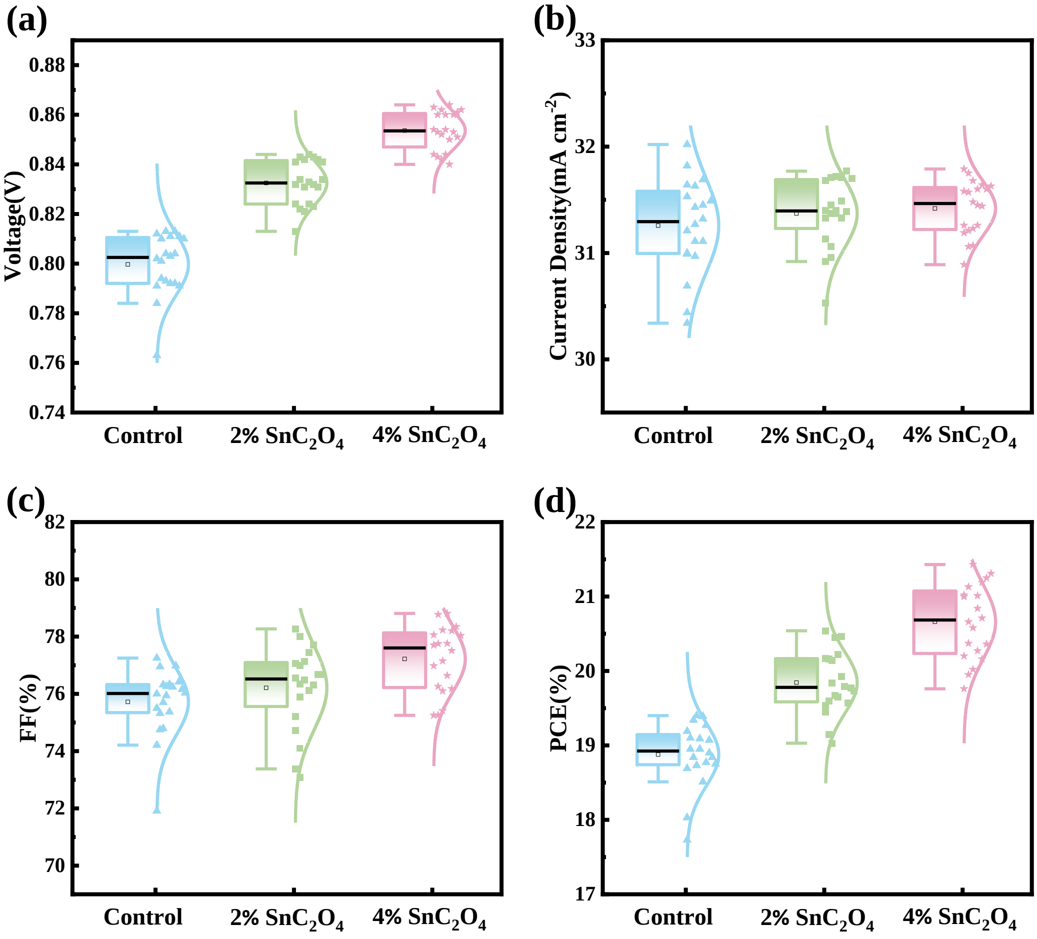

Figure S13. The photovoltaic parameter distribution of 20 cells for same condition of the control, 2% SnC_2O_4 and 4% SnC_2O_4 treated device.

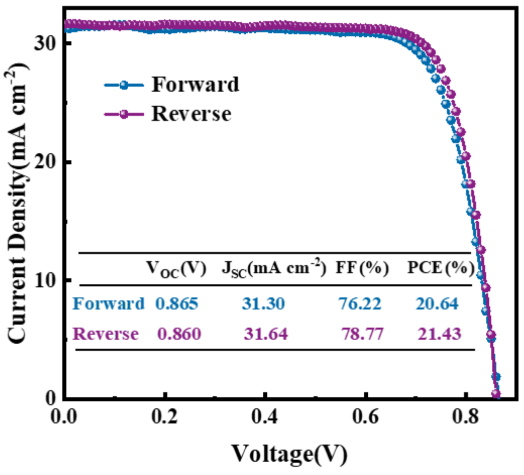

Figure S14. Forward and reverse J-V curves s of the Champion Cell.

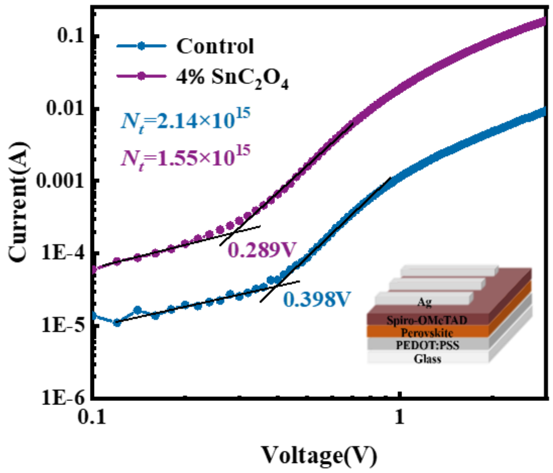

Figure S15. Space charge limited current measurement of the control and 4% SnC_2O_4 treated device.

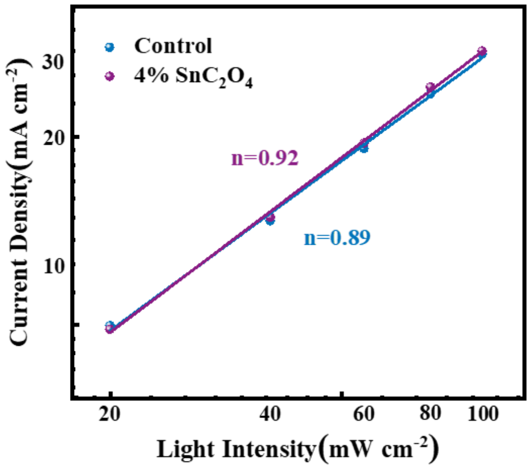

Figure S16. Light intensity dependence of J_{SC} for devices based on different perovskite films.

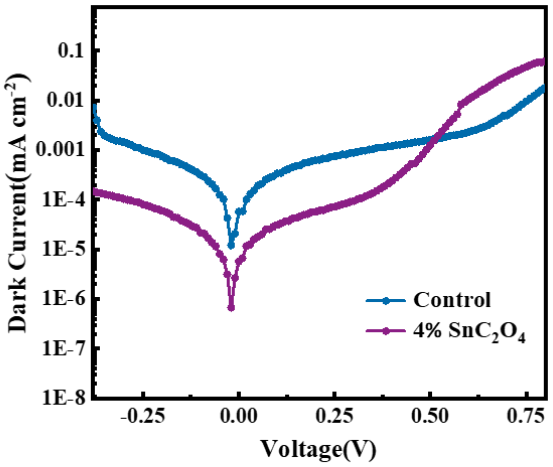

Figure S17. The dark J-V curves of devices based on control and 4% SnC_2O_4 devices.

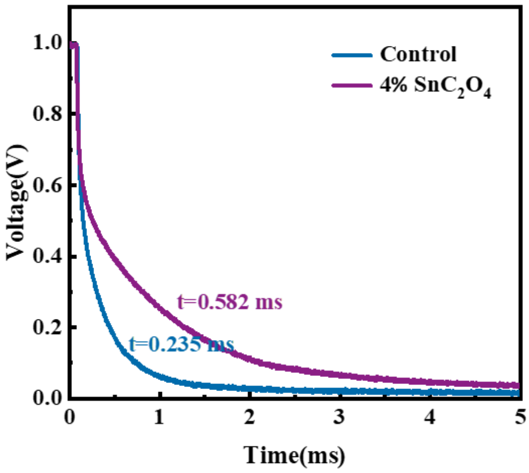

Figure S18. TPV of control and 4% SnC_2O_4 devices.

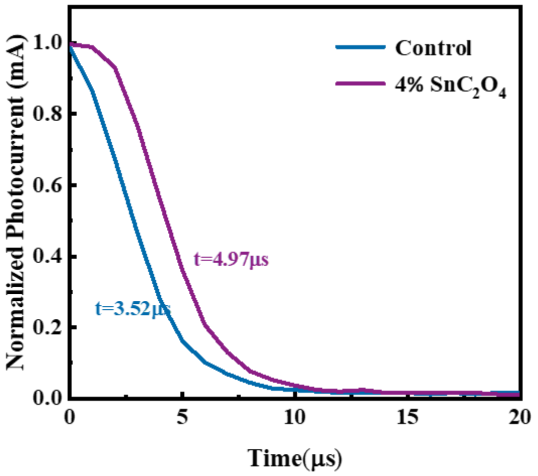

Figure S19. TPC of control and 4% SnC_2O_4 devices.

	E_{cutoff} [eV]	E_{onset} [eV]	E_V [eV]	E_C [eV]	E_g [eV]
**Control**	**16.66**	**0.87**	**5.40**	**4.14**	**1.26**
**4% SnC_2O_4**	**16.72**	**0.84**	**5.37**	**4.11**	**1.26**

Table S1. Detailed parameters of the energy band structure.

	V_{OC} [V]	J_{SC} [mA cm^{-2}]	FF [%]	PCE [%]
**Control**	**0.81**	**31.32**	**76.24**	**19.34**
**2% SnC_2O_4**	**0.83**	**31.33**	**78.01**	**20.29**
**4% SnC_2O_4**	**0.86**	**31.64**	**78.77**	**21.43**

Table S2. The optimal photoelectric parameters of control, 2% SnC_2O_4 and 4% SnC_2O_4 doped perovskite devices.
